# Pre-gestational diabetes: benefits and barriers to attendance at pre-pregnancy clinics

**DOI:** 10.1007/s11845-025-04037-9

**Published:** 2025-08-15

**Authors:** Cathriona Murphy, Linda Culliney, Sinead Whelan, Louise O’Mahony, Ashling Kennedy, Michelle Sugrue, Adrianne Wyse, Oratile Kgosidialwa, Mairead O’Riordan, Antoinette Tuthill

**Affiliations:** 1 School of Medicine, University College Cork, Cork, Ireland; 2 Department of Dietetics, Cork University Hospital, Cork, Ireland; 3 Department of Diabetes and Endocrinology, Cork University Hospital, Cork, Ireland; 4 Department of Obstetrics and Gynaecology, Cork University Maternity Hospital, Cork, Ireland

**Keywords:** Attendance, Barriers, Diabetes mellitus, Facilitators, Pre-gestational, Pregnancy, Pre-pregnancy clinic

## Abstract

**Background:**

Pre-gestational diabetes mellitus increases the risk of adverse pregnancy outcomes. Pre-pregnancy clinics are a well-established and cost-effective way of reducing pregnancy complications.

**Aims:**

This study aimed to review outcome differences between women with pre-gestational diabetes who did/did not attend pre-pregnancy clinics and to evaluate barriers to attendance.

**Methods:**

1. A retrospective study examined data from all women with pre-gestational diabetes who received obstetric care in Cork University Maternity Hospital 2015–2019.
2. A cross-sectional survey of women attending antenatal clinics during the study period. A telephone questionnaire was completed to understand facilitators and barriers to pre-pregnancy attendance.

**Results:**

Two hundred women were included retrospectively: 65.5% with Type1 diabetes, and 30.5% with type 2 diabetes. Only 26% (52) attended pre-pregnancy clinics. Although there were no differences in miscarriage rate, congenital anomaly, mode of delivery or neonatal intensive care unit admission between groups, the mean birth weight of babies born to women who attended pre-pregnancy clinics was less than those who did not attend (3294.5 ± 753.0 g vs. 3598.8 ± 802.8 g; *p* = 0.02).

Twenty-eight women were included cross-sectionally, nine of whom attended pre-pregnancy clinics. All who attended found it useful. Eight participants proposed a hybrid clinic model to optimise future service engagement. Increasing awareness was advocated by many participants.

**Conclusion:**

Attendance at pre-pregnancy clinics is low. Lack of awareness is the greatest barrier reported to attendance. To optimise perinatal outcomes, efforts are required to increase attendance; this may be facilitated by increasing awareness of these clinics, as suggested by the women themselves.

## Introduction

Women with pre-gestational (PG) diabetes and their infants are at increased risk of operative delivery, neonatal morbidity and congenital anomalies [1, 2]. It is well established however that optimising maternal glycaemic control significantly reduces the risk of miscarriage, congenital malformation, stillbirth and neonatal mortality [1, 3].

The National Institute for Health and Care Excellence (NICE) outlines the importance of optimising glycaemic control, cessation of teratogenic medications and the use of folic acid at least 12 weeks prior to conception in women with PG diabetes [4]. Ensuring women attend pre-pregnancy services to educate and assist them to achieve these targets is feasible and cost effective [5].

The prevalence of pregnancies complicated by diabetes is increasing globally [6, 7]. From 1998 to 2013, using the Scottish Morbidity Record, the number of pregnancies complicated by diabetes increased by 44% in type 1 diabetes (T1) and 90% in type 2 diabetes (T2) [7]. The higher increase in women with type 2 diabetes is in parallel with increasing obesity and maternal age. The substantial rise in the number of women with PG diabetes in recent years demonstrates the importance of encouraging attendance at pre-conceptual services for these women.

A study conducted in Ireland from 2005 to 2010 highlighted how implementing change in the provision of pre-pregnancy services can have beneficial effects on women with PG diabetes in pregnancy [8]. This study showed significant improvement in pre-pregnancy HbA1c and overall pregnancy outcomes following a twofold increase in attendance at pre-pregnancy clinics (PPC). Unfortunately, despite pre-pregnancy care and education being a proven method of improving pregnancy outcomes, attendance at PPC in Ireland remains low, with only 30% of women attending [9]. One Irish study showed that only 15.9% of women were adequately prepared for pregnancy [9]. Similarly in the UK, studies demonstrated that a minority of women with T1 and T2 diabetes are meeting pre-pregnancy targets and are optimally prepared for pregnancy (14% vs 37% respectively) [10].

Although it is well established that engagement with PPC is poor, there is a paucity of data regarding the factors that determine whether a woman will attend PPC.

### Aim

The aim of our study was firstly to retrospectively review the clinical outcomes of women who attended our diabetes antenatal services over a 5-year period to determine differences between those who did/did not attend PPC.

In addition, using a cross-sectional survey, we aimed to evaluate barriers and facilitators to PPC attendance in women currently attending our diabetes antenatal clinic in order to mould a service that would be increasingly attended by this growing population.

## Methods

### Study design, study site, and population

This study, was conducted in Cork University Maternity Hospital (CUMH) from August 2021 to July 2022. CUMH is one of 20 maternity centres in Ireland, with 126 maternal beds and is a tertiary referral centre for obstetric care in the South/South-West of Ireland. One of the largest cohorts of Irish women with pre-existing diabetes attend CUH and CUMH for their antenatal diabetes care. Ethical approval for this study was granted by the Clinical Research and Ethics Committee of Cork Teaching Hospitals (CREC) in July 2021 [11].

In the first part of this study, we retrospectively examined data from all 200 women with PG diabetes who received their obstetric care in CUMH between 2015 and 2019. Participants’ details including demographics, clinical and laboratory data were extracted from their hospital electronic health records (Maternal & Newborn Clinical Management System, i.Laboratory and i.Clinical Manager). Data including participant’s age, BMI, ethnicity, co-morbidities, diabetes-related complications, pregnancy outcomes, medication use and PPC attendance was collected.

The second part of this study, a cross-sectional survey, included women with PG diabetes attending the antenatal diabetes clinic in CUMH during the study period. After informed written consent was obtained, women satisfying the inclusion criteria were invited to participate. The inclusion criteria were pregnant women with PG diabetes aged 18 years or above. Women with gestational diabetes or those who were unable/unwilling to provide consent were excluded from the study. A questionnaire (Appendix 1) was developed by members of the multi-disciplinary team (diabetologists, obstetricians, diabetes midwives, diabetes nurses and dieticians) involved in care of women with PG diabetes during pregnancy to enquire about factors which would be more likely to increase a woman’s attendance at PPC and identify any barriers to same. The questionnaire consisted of 15 questions. This study was performed during the COVID-19 pandemic when social distancing measures were in place; therefore, surveys were completed over the telephone to minimise face-to-face contact. Participant demographic data, obstetric history, pregnancy outcomes and diabetes history were obtained from hospital electronic health records.

### Statistical analysis

Statistical analysis was carried out using the IBM SPSS Programme Version 28.0. Data was summarised using descriptive statistics. For continuous variables, the mean and standard deviation were reported for normally distributed data. Median and minimum to maximum were used to summarise non-normally distributed data. Categorical data was reported as counts and percentages. Logistic regression was used to assess the associations between attendance at PPC and pregnancy outcomes such as miscarriage, mode of delivery, NICU admission and birth weight. Differences were considered to be statistically significant if *p*-value was < 0.05.

## Results

### Retrospective study

Participant baseline characteristics are shown in Table 1. Of the 200 women reviewed, 131 (65.5%) had T1 diabetes, 61 (30.5%) had T2, six had monogenic diabetes (MODY), one had pancreatitis-induced diabetes and one had transplant-associated diabetes.

**Table 1:** Demographic data of women with T1 and T2 diabetes pre-dating pregnancy

	All patients *N* = 200	T1 diabetes *N* = 131	T2 diabetes *N* = 61	*p*-value
Age, mean ± SD (years)	33.9 (5.6)	32.6 ± 5.5	36.7 ± 5.2	< 0.001
Caucasian ethnicity, *n* (%)	182 (91.0%)	130 (99.0%)	45 (73.8%)	< 0.001
BMI, mean ± SD (kg/m^2^)	28.8 ± 6.4	26.4 ± 4.2	34.5 ± 6.9	< 0.001
BMI category, *n* (%)				
< 24.9 kg/m^2^	58 (29.0%)	50 (38.1%)	4 (6.6%)	< 0.001
25–30 kg/m^2^	61 (30.5%)	46 (35.1%)	11 (18.0%)	< 0.001
> 30 kg/m^2^	62(31.0%)	21 (15.7%)	41 (67.2%)	< 0.001
Duration of diabetes, mean ± SD (years)	12.2 ± 9.1	15.7 ± 8.9	4.7 ± 3.7	< 0.001
Gravidity, (median [min–max])	2.0 [1–4]	2.0 [1–3]	2.5 [1–4]	< 0.001
Parity (min–max)	1 [0–3]	1 [0–3]	1 [0–3]	0.003
Retinopathy, n(%)	24 (12.0%)	20 (15.3%)	0	< 0.001
Ischaemic heart disease, *n* (%)	1 (0.5%)	0	1 (1.6%)	0.341
Hypertension, *n* (%)	28 (14%)	16 (12.2%)	12(19.6%)	0.256
Microalbuminuria, *n* (%)	16 (8%)	13 (9.9%)	2 (3%)	0.003
PPC Attendance, *n* (%)	52 (26%)	37 (28.2%)	13 (21.3%)	0.17
5 mg folic acid use at LMP, *n* (%)	94 (47%)	68 (51.9%)	25 (41%)	0.088
Statin use at LMP, *n* (%)	2 (1%)	2 (1.5%)	0	0.449
ACEi/ARB usage at LMP, *n* (%)	18 (9%)	11 (8%)	7 (11.4%)	0.432
Pre-pregnancy HbA1c, mean ± SD mmol/mol [%])	63 ± 20 [7.9 ± 4.0]	65 ± 20 [7.5 ± 3.9]	58 ± 19 [8.1 ± 4.0]	0.01

*BMI* body mass index, *LMP* last menstrual period, *ACEi* angiotensin converting enzyme inhibitor, *ARB* angiotensin receptor blocker

Women with T1 diabetes were younger (*p* < 0.001), more likely to be Caucasian (*p* < 0.001), had a longer duration of diabetes (*p* < 0.001) and a higher burden of microvascular complications when compared to women with T2. A higher proportion of women with T2 diabetes were obese when compared to women with T1 (*p* < 0.001).

### Pregnancy preparedness

Of the 52 women (26.0%) who attended PPC, 37, 13 and two had T1 diabetes, T2 and ‘other’ types of diabetes respectively. Women with T2 diabetes had a lower HbA1c prior to pregnancy than women with T1 (*p* < 0.05). Ninety-four (47.0%) women were on high dose folic acid prior to pregnancy (Table 1).

### Pregnancy outcomes

Outcomes of women who attended PPC compared to those who did not are shown in Table 2. The mean birth weight of babies born to women who attended PPC was less than those who did not attend (3294.5 ± 753.0 g vs. 3598.8 ± 802.8 g; *p* = 0.02).

**Table 2:** Outcomes of women who attended PPC vs those who did not attend PPC

	Attended clinic	Did not attend clinic	*p-value
	*N* = 52	*N* = 148	
	*N* (%)	*N* (%)	
Pregnancy outcome			0.47
Live birth, *n* (%)	43 (83%)	122 (83.6%)	
Miscarriage < 20 weeks, *n* (%)	8 (15%)	20 (13.7%)	
Elective termination, *n* (%	0	3 (2%)	
Stillbirth (> 24 weeks), *n* (%)	0	1 (0.7%)	
Neonatal death, *n* (%)	1 (2%)	0	
Congenital anomaly	2 (4%)	6 (5%)	0.32
Mode of delivery	*N* = 43	*N* = 118	0.78
Spontaneous vaginal delivery	11 (26%)	28 (24%)	
Ventouse vaginal delivery	1 (2%)	6 (5%)	
Forceps vaginal delivery	2 (5%)	3 (3%)	
Elective caesarean section	13 (30%)	44 (37%)	
Emergency caesarean section	16 (37%)	37 (31%)	
Birth weight (g)	*N* = 44	*N* = 121	0.09
≤ 2500	4 (9%)	12 (10%)	
≥ 4001	4 (9%)	24 (20%)	
≥ 4500	2 (5%)	16 (13%)	
NICU/SCBU admission	23 (52%)	54 (45%)	0.41
Yes			
Antenatal maternal hospitalisation	*N* = 51	*N* = 143	0.97
Yes	28 (55%)	79 (55%)	
Preterm birth*, *n* (%)	*N* = 49	*N* = 129	0.27
	17 (35%)	34 (26%)	
	Mean ± sd	Mean ± sd	p-value
Birth weight (g)	3294.5 ± 753.0	3598.8 ± 802.8	0.02
5-min Apgar, median (min–max)	10 (7–10)	9 (4–10)	0.08
Pre-pregnancy HbA1c (mmol/mol [%])	59 ± 15 [7.6 ± 3.5	64 ± 21 [8.0 ± 4.1]	0.09
Trimester 1 HbA1c	55 ± 16 [7.2 ± 3.6]	58 ± 17 [7.4 ± 3.7]	0.16
Trimester 2 HbA1c	43 ± 10 [6.1 ± 3.1]	45 ± 11 [6.3 ± 3.2]	0.10
Trimester 3 HbA1c	44 ± 12 [6.2 ± 3.2]	46 ± 11 [6.3 ± 3.1]	0.19

^*^Preterm birth: less than 37 weeks’ gestation at birth

Of the women who attended PPC, 10 (20%) had babies above the 95th centile when adjusted for gestational age in comparison with 38 (26%) of the babies of mothers who did not attend (*p* = 0 0.092).

### Cross-sectional survey

**Fig. 1:**
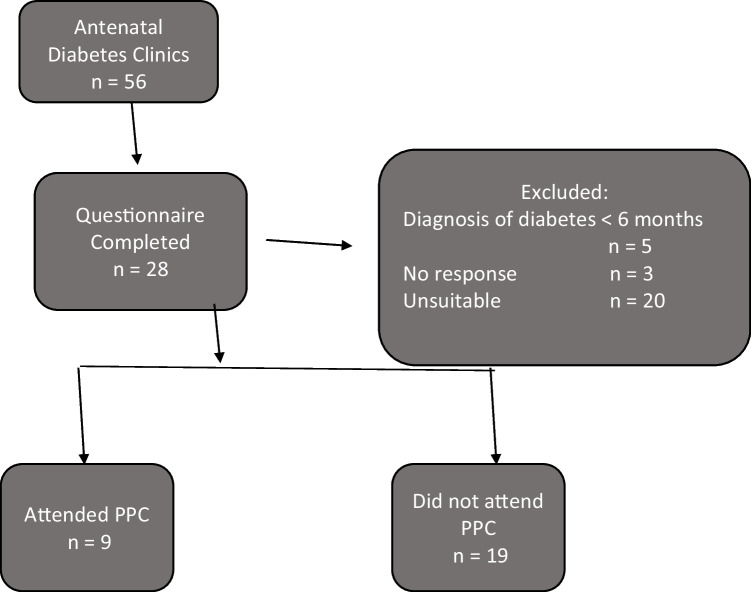
Flowchart depicting the patient recruitment process

Of the 56 women who attended antenatal diabetes clinics, 20 were considered unsuitable for participation following multidisciplinary team (MDT) discussion for reasons such as language barriers, a history of poor clinic attendance previously, concern regarding significant underlying mental health comorbidities or recently moved to area; three did not respond, and five had a diabetes diagnosis of under 6 months (Fig. 1).

Of the remaining 28 women included in this study, only nine (32%) attended PPC, although 16 reported they were aware that PPC clinics were held in our centre prior to conceiving. Four of the 19 pregnancies in those who did not attend PPC were unplanned. Nine of those who did not attend reported that they would have attended had they been aware of same (Table 3).

**Table 3:** Cross-sectional survey

	All women (N=28)	Attended PPC (N=9)	Did Not attend PPC (N=19)
Mean age (years)	33.7 ± 4.5	32.9 ± 4.6	33.1 ± 4.5
Mean BMI at booking visit (Kg/m^2^)	31.5 ± 7.2	31.4 ± 7.3	31.5 ± 7.1
T1DM	18 (64%)	9 (100%)	9 (47%)
T2DM	10 (36%)	0	10 (53%)
Women who attended PPC	9 (32%)	9 (32%)	19 (68%)
Women aware of PPC prior to conceiving	16 (57.1%)	9 (100%)	7 (37%)
Women who would have attended PPC had they been aware	9	NA	9 (47.3%)
Folic acid pre-conception	21 (75%)	9 (100%)	12 (63.1%)
Folic Acid post-conception	28 (100%)	9 (100%)	19 (100%)

### Pre-pregnancy clinics: barriers to attendance

Of 10 women who reported that they would not have been able to attend PPC, even if aware of same, practical constraints such as inconvenient time was a factor for 3. Five of those who did not attend felt that PPC attendance was not necessary as they considered they had good glucose control already (Table 4). All of the women who attended found the information provided useful.

**Table 4:** Pre-pregnancy clinics: barriers to attendance

	(Non-attendees *N* = 19)
Barrier to PPC	
Lack of awareness regarding PPC	12 (63%)
Practical constraints, e.g., insufficient time or inconvenient time 32.96	3 (16%)
Felt PPC not necessary, e.g., believed they had good glucose control	5 (26%)
	(Attendees *N* = 9)
Referral source for PPC	
Consultant endocrinologist	4 (44%)
Diabetes nurse specialist (DNS)	4 (44%)
Diabetes Ireland contact	1 (11%)
General practitioner	
Perceived usefulness/benefit of PPC	
General information useful	9 (100%)
Specific information on blood glucose	6 (67%)
Reassurance from extra support	5 (56%)
	All women (*N* = 28)
Preferred clinic format	
Face-to-face	11 (39%)
Virtual	9 (32%)
Hybrid (both)	8 (29%)
First appointment face-to-face and virtual thereafter	3 (10%)
Preferred appointment duration	
15–30 min	16 (57%)
> 30 min	3 (10%)
Preferred clinic frequency	
Weekly	1 (3%)
Fortnightly	12 (43%)
Monthly	10 (36%)
Preferred clinic providers	
Diabetes nurse specialist (DNS) only	11 (39%)
DNS and dietitian	8 (29%)
DNS and dietitian and doctor	9 (32%)
Awareness and outreach preferences	
Reputable healthcare source (e.g., GP, DNS, diabetic clinics, medical leaflets)	11 (39%)
Social media	12 (43%)
Multichannel outreach (including all the above methods)	6 (21%)

When the 28 women were asked about what style of clinic they felt would be increasingly attended, 11 (39%) maintained that face-to-face clinics was their preference; nine (32%) preferred a virtual clinic and eight (29%) felt that a combination of both face-to-face and virtual appointments would be ideal (Table 4).

Optimal duration of appointments desired was 15–30 min, with attendance on a fortnightly basis deemed to be the most practical.

Only one felt that attending on a weekly basis would be feasible, due to practical constraints such as childcare and absences from work.

Lack of awareness was the most common factor reported limiting attendance at PPC. When asked about where best to target these women to inform them of this service, 12 of the women were of the opinion it was appropriate to raise awareness via social media in addition to other channels, in order to optimise the public reach.

## Discussion

This study was conducted to initially retrospectively review differences in outcomes between women with pre-gestational diabetes who did and did not attend PPC.

Of the 200 participants examined, a majority (65.5%) had T1 diabetes, were younger and had a longer duration of diabetes than those with T2*.* This is in keeping with other studies which suggest that 70% women with pre-gestational diabetes in Ireland have T1 and 30% T2. The changing population demographic however is likely to increase the proportion of women attending pregnancy with T2 diabetes in future years [12]. In keeping with this previous study, more women with T1 diabetes attended PPC and were taking 5 mg folic acid pre-pregnancy [12]. The existing evidence suggests that attendance at PPC results in superior glycaemic control and pregnancy outcomes. In this study, women who attended PPC had a lower HbA1c throughout all trimesters of pregnancy. The babies of women who attended PPC also had a lower birthweight when compared with the babies of women who did not attend PPC.

Secondly, we aimed to evaluate factors which could make PPC more accessible to facilitate attendance at the clinics by obtaining data in a cross-sectional manner on women with pre-gestational diabetes who were attending diabetes antenatal clinics. Despite evidence demonstrating the significant benefit of attending PPC, a previous study from the Atlantic Dip group 2008–2010 reported 52% of women had attended PPC [8]. In our cohort, however, only nine of 28 women attended PPC. We found that a lack of awareness was the most significant barrier to attendance reported in this cohort of women, with 43% advising that they were not aware of the existence of the PPC service. Furthermore, similar to O’Higgins et al., practical constraints such as work obligations and childcare were major barriers reported to attendance at PPC [13]. The participants also expressed concerns regarding planning pregnancy and physicians placing too much emphasis on negative outcomes and complications resulting from pre-gestational diabetes in pregnancy.

An important consideration based on this study is instrumenting an effective referral pathway for women with PG diabetes to pre-pregnancy care and expanding the reach and awareness of primary care physicians about this service. Our study demonstrated that only 16.7% of women who attended PPC were referred by their general practitioner. A significant proportion of care for women with pre-gestational diabetes, particularly T2 diabetes, takes place in a primary care setting; therefore, a stronger emphasis needs to be placed on the importance of pre-conception care and primary care physicians need to be counselled on the availability of the services available to these women and must place a stronger emphasis on the importance of pre-conception care [14].

An active effort using social media and improved advertising of this service at diabetes clinics and support groups need to be undertaken in order to establish an increased awareness and knowledge of PPC. During the COVID-19 pandemic, the fruits of online information and awareness were widely established and use of social media platforms can positively influence public awareness and behaviour [15]. In this study, where participants were aged between 22 and 40 years, and are highly engaged online, expanding beyond traditional methods of communication would definitely augment the awareness campaign.

In addition, in order to tackle some of the practical constraints raised by participants, the introduction of a hybrid model was suggested by a number of participants: 43% of women felt that attending on a fortnightly basis for up to 30 min was most practical. However, many felt that attending the first clinic appointment in person and allowing subsequent visits to be done virtually would greatly ease the pressure on these women. A study, conducted by Nerpin et al. in Sweden, examined the effect of virtual diabetes care in young patients with T1 diabetes. It evaluated their glycaemic control, diabetes-related complications and the burden of T1 diabetes on their lives. This study found that virtual care had the potential to reduce suboptimal glycaemic control and the burden of diabetes on these patient’s lives [16]. This is an important consideration when contemplating the service model, which should be implemented for these women. It may assist in overcoming the practical burden of frequently attending clinic whilst continuing to offer advice and supportive management to these women.

## Limitations

This study has some limitations. One phase of this study was retrospective, which introduces the potential for missing data and exclusion of valid participants because of a lack of data availability. There is also the risk of data omission for example, unavailable laboratory results, loss due to transfer of care or patient exclusion due to lack of records of miscarriages and early pregnancy losses.

Prospectively, this study was carried out in a single institution with a small sample size. Whilst one of the largest cohorts of Irish women with pre-existing diabetes attend CUH and CUMH for their antenatal diabetes care, unfortunately, a significant number of women were not eligible or declined participation in the study. As the questionnaire was conducted over the phone, non-response was also a limitation in this study.

Although pregnancy is usually associated with increased attendance, even in those who may otherwise miss appointments, there are still a cohort of women who do not attend for diabetes care during pregnancy. One of the limitations of this study is that the cross-sectional part only included women who were currently attending for antenatal diabetes care, hence may not have determined barriers limiting attendance of other women with PGM to these services.

## Conclusion

In summary, it is clear that there is still a large discrepancy in the number of women attending PPC despite a strong evidence base advocating for the service. Attendance at PPC is one of the critical modifiable risk factors for improved maternal and foetal outcomes. Women with pre-gestational diabetes need support and reassurance. They need advice to optimise their glycaemic control, dietary habits and ensure medication safety prior to pregnancy.

In our opinion, there is the potential for further research to be conducted in this area. In particular, completing a quality improvement initiative to re-assess engagement with the service after implementation of some of the critical suggestions made by these women would be of value in ensuring an enhanced pre-pregnancy service.

Unfortunately, lack of awareness and practical constraints are the biggest barriers reported to attendance at PPC. Therefore, the possibility of introducing a hybrid model of service provision combined with a multimodal campaign to increase public awareness may facilitate increased attendance at these clinics.

## Data Availability

The data that support the findings of this study are available on request from the corresponding author, AT.

## Appendix 1

See Table 5.

**Table 5:** Pre-pregnancy clinic attendance questionnaire

Pre-Pregnancy Attendance Questionnaire	
Were you aware of PPC prior to your pregnancy?	Yes/No
If no, would you attendance had you been aware?	Yes/No
If no, why not?	Inconvenient time Good glucose control
	Inconvenient location Previously attended
	Other
Where did you attend PPC?	
Where did you first hear about PPC?	General Practitioner Diabetes Nurse Specialist
	Consultant Endocrinologist Dietician
	Community Structured education course
If you attended, who referred you?	General Practitioner Diabetes Nurse Specialist
	Consultant Endocrinologist Dietician
	Community Structured education course
Did you find it useful?	Yes/No
What information was most useful?	Blood glucose Targets Diet Information
	Medication review Information on obstetric service
	Other
What kind of clinic would you prefer?	Face to face Virtual Consultation
	Both Other
Who would you like to meet with at the clinic?	Diabetes Nurse Specialist Dietician
	Doctor
How long would you like the clinic to be?	<15 minutes > 30 minutes
	15-30 minutes
How frequently would you like an appointment?	Weekly Fortnightly
	Monthly Every 2 months
	Every 3 months
Where would you like these clinics to take place?	Diabetes Day Care Unit, CUH Online
	Community Setting Maternity Hospital
	G.P. Practice
Where should you receive information about these clinics?	Diabetes clinics Retinal Screening
	G.P. appointments Other
How could we raise awareness about PPC?	Social Media G.P. Visits
	Leaflets at clinics Leaflets at support groups
	Other
Are you taking folic acid?	Yes/No
When did you start taking folic acid?	Pre-pregnancy. After positive test
What dose of folic acid are you taking?	400 micrograms 5mg
Are you currently taking any other medications during this pregnancy?	
Did you attend fertility services prior to becoming pregnant?	Yes/No
If yes, did anybody discuss your diabetes care/targets with you?	Yes/No

PPC Pre-Pregnancy Clinic, G.P. General Practitioner
